# DeepCNet: A multimodal deep learning model for predicting cell type-specific gene expression and promoter-enhancer interactions from single-cell multiome data

**DOI:** 10.21203/rs.3.rs-8716015/v1

**Published:** 2026-09-03

**Authors:** Weijia Jin, Fengdi Zhao, Xin Ma, Qing Lu, Karyn A. Esser, Ramon C. Sun, Li Chen

**Affiliations:** 1 Department of Biostatistics, University of Florida, Gainesville, FL, USA; 2 Department of Physiology and Aging, University of Florida, Gainesville, FL, USA; 3 Claude D. Pepper Older Americans Independence Center, University of Florida, Gainesville, FL; 4 Department of Biochemistry and Molecular Biology, University of Florida, Gainesville, FL, USA; 5 Center for Advanced Spatial Biomolecular Research, University of Florida, Gainesville, FL, USA; 6 Evelyn F. and William L. McKnight Brain Institute, University of Florida, Gainesville, FL, USA; 7 Cancer Institute, University of Florida, Gainesville, FL, USA

## Abstract

Understanding promoter-enhancer (PE) interactions is essential for elucidating gene regulation mechanisms. Although existing computational methods leverage DNA sequence to predict gene expression, many were developed primarily for bulk-level genomic data and emphasize predictive accuracy rather than regulatory inference. Here, we propose DeepCNet, a multimodal deep learning framework that predicts cell type-specific gene expression and infers cell type-specific PE interactions, enabling the construction of transcription factor gene regulatory networks (TF-GRNs) from single-cell multiome data. Evaluated on two single-cell multiome datasets from blood and brain tissues, DeepCNet consistently outperforms state-of-the-art methods in predicting cell type-specific gene expression. Importantly, PE interactions inferred by DeepCNet showed greater concordance with orthogonal chromatin interaction and CRISPR perturbation data than competing approaches. Analysis of cell type–specific GRNs identified TF hubs and highlighted both cell type–specific and shared regulatory programs. DeepCNet further extends to two-condition single-cell multiome studies, enabling the identification of differential PE interactions and characterization of TF-GRN alterations across conditions. As an illustrative application, we applied DeepCNet to single-cell multiome data from one Alzheimer’s disease (AD) and one healthy control sample, identifying candidate differential PE interactions at AD risk loci and candidate TF hubs associated with AD-related pathways. Together, these results demonstrate the potential of DeepCNet to characterize transcriptional regulatory programs and cooperative TF interactions underlying cell type- and condition-specific gene expression.

## Introduction

Evaluating how noncoding DNA determines gene expression in different cell types remains a fundamental challenging problem in genomics, with important downstream applications such as inferring cell type-specific PE interactions. Recently, “Sequence-to-function” deep learning approaches have been developed to leverage DNA sequence information in the neighborhoods of transcription start site (TSS) for gene expression prediction. For example, ExPecto uses DNA sequence spanning ±20 kb around each TSS as input to a multi-task convolutional neural network for predicting 2002 chromatin features, which are subsequently regressed to estimate gene expression [1]. Enformer further extends the input DNA sequence to 196 kb and adopts a multi-task, multi-target framework to jointly predict genome-wide profiles of gene expression (CAGE), chromatin accessibility (DNase/ATAC), and ChIP-seq signals for histone marks and transcription factor binding [2]. Both ExPecto and Enformer are developed in bulk tissue or independent cell type settings with training set aggregated from large-scale consortia such as ENCODE and Roadmap Epigenomics Project [3, 4].

Recent advances in single-cell technologies now enable the profiling of gene expression (single-cell RNA-seq), chromatin accessibility (single-cell ATAC-seq), and their joint measurement (single-cell multiome-seq) at single-cell resolution across diverse cell types. These developments create new opportunities to link gene regulatory regions to gene expression on single-cell and cell type-specific level. However, the unique characteristics of single-cell data pose both challenges and opportunities for predictive modeling. Cell types are often correlated through hierarchical relationships defined by lineage or ontology, which can be exploited to improve cell type–specific modeling of gene expression and regulatory activity by borrowing information from related cell types [5, 6]. Moreover, as cis-regulatory elements (CREs) such as promoters and enhancers identified from ATAC peaks, are particularly informative for gene regulation, restricting sequence inputs to CREs can substantially reduce feature dimensionality and model complexity, facilitating more efficient and interpretable training of sequence-based models. Accordingly, “sequence-to-function” deep learning frameworks have been extended into single-cell or cell type-specific settings. Building upon Enformer, Borzoi aims to predict RNA-seq coverage from DNA sequence and seq2cells generates DNA sequence embeddings from ~200 kb regions centered on TSSs and predicts gene expression across all cells of a single-cell dataset [7, 8]. Although EPInformer, another transformer-based approach, does not explicitly model cell type heterogeneity, it predicts gene expression by integrating regulatory sequences, epigenomic signals, and chromatin contacts, with one important downstream application for inferring PE interactions for one given cell type (e.g. K562, GM12878)[9]

Despite these advances, most existing approaches primarily emphasize on gene expression prediction, while gene regulation inference, particularly cell type–specific PE interactions and GRN reconstruction, has not been the central focus. To address this gap, we propose DeepCNet, a multimodal deep learning framework for inferring cell type-specific PE interactions and constructing GRNs using single-cell multiome data. DeepCNet consists of two complementary modes. DeepCNet-S is designed to infer cell type-specific PE interactions under a single biological condition and to construct corresponding GRNs from a given single-cell multiome dataset, whereas DeepCNet-D performs joint modeling of two-condition single-cell multiome data (e.g., disease versus healthy control) to infer differential PE interactions and condition-specific GRN rewiring. To the best of our knowledge, this is among the first efforts to systematically model differential PE interactions and GRNs in a single-cell multiome framework. We evaluated DeepCNet on two representative single-cell multiome datasets from blood and brain tissues. DeepCNet consistently outperforms existing approaches in predicting cell type-specific gene expression, while its inferred PE interactions show higher concordance with orthogonal chromatin interaction and CRISPR perturbation data. Building upon the inferred PE interactions, DeepCNet constructs TF-GRNs to identify TF-hubs and TF-TF interactions that regulate target genes involved in cell type- and disease relevant transcriptional programs. Notably, DeepCNet shows an illustration application for identifying candidate differential PE interactions between one AD and one healthy control at key risk loci and candidate rewired TF hubs enriched in AD-associated pathways.

## Results

### Overview of DeepCNet

DeepCNet is a multimodal deep learning framework designed for predicting cell type-specific gene expression, inferring PE interaction and constructing TF-GRN by leveraging single-cell multiome data, which jointly profiles RNA expression and chromatin accessibility within the same cells. DeepCNet integrates DNA sequence features with chromatin accessibility at ATAC peaks representing candidate enhancers near TSSs through a flexible architecture that explicitly models their regulatory relationships. DeepCNet consists of two complementary variants, DeepCNet-S and DeepCNet-D. DeepCNet-S focuses on inferring PE interactions and TF-GRNs, whereas DeepCNet-D jointly models two biological conditions to identify candidate differential PE interactions and characterize alterations in TF-GRNs (Figure 1). DeepCNet-S consists of four key components: (i) Sequence encoder. This module transforms DNA sequences from gene promoters and their nearby ATAC peaks (i.e. within ±100 kb of TSS) into high-dimensional, tissue- and cell type-aware epigenomic features using a pretrained sequence-to-function model (i.e. ExPecto[1]). For genes with fewer than the predefined number of peaks, padding is applied, and all peaks are resized to a uniform length. Additional ATAC-derived features, including peak-to-TSS distance and chromatin accessibility, are integrated with the sequence-based epigenomic features to form the encoder output. (ii) Chromatin interaction module. Taking sequence encoder output as input, this module models PE interactions using an multi-head attention (MHA) encoder. (iii) Cross-cell type autoencoder. This component generates a joint gene embedding by integrating cell type-specific gene embeddings from gene activity while accounting for cell type relationships, thereby facilitating information sharing across related cell types. (iv) Gene expression prediction module. This module integrates the embeddings learned by the chromatin interaction module and cross-cell type autoencoder to predict cell type-specific gene expression.

DeepCNet-D extends DeepCNet-S to detect cell type-specific differential PE interactions, thereby enabling the identification of condition-specific alterations in TF-GRNs across biological conditions. Specifically, DeepCNet-D adopts a multitask learning framework that jointly models two conditions using shared DNA sequence features from a common set of ATAC peaks together with condition-specific chromatin accessibility. Gene expression is simultaneously predicted for both conditions. To capture differential regulatory mechanisms, DeepCNet-D employs a shared MHA encoder with learnable condition-aware parameters, enabling the model to distinguish PE interactions that are shared across conditions from those that are condition-specific. Collectively, DeepCNet provides a unified deep learning framework that integrates sequence context with chromatin accessibility and cell type relationships derived from single-cell multiome data to infer cell type–specific PE interactions, reconstruct TF-GRNs, and characterize their rewiring across biological conditions.

### DeepCNet improves cell type–specific gene expression prediction

To evaluate DeepCNet for cell type-specific gene expression prediction, we adopted a cross-chromosome validation strategy to provide an independent assessment of model performance, with chromosomes partitioned into training, validation, and testing sets (Methods). DeepCNet integrates DNA sequence-derived features, chromatin accessibility, and peak-to-TSS distance to predict gene expression at the cell type level. We benchmarked DeepCNet against EPInformer, ExPecto, and two variants of seq2cells (seq2cells-cell and seq2cells-celltype; Methods) using 12-fold cross-chromosome validation [1, 7, 9]. Given the substantial computational requirements associated with model training, we additionally compared DeepCNet with Enformer and Borzoi using a single cross-chromosome fold (Methods) [2, 8]. Prediction performance was evaluated using the Pearson correlation coefficient (R) and root mean squared error (RMSE). Benchmarking was performed on two representative single-cell multiome datasets derived from blood and brain tissues.

Figure 2A summarizes prediction performance, measured by Pearson correlation (R) and root mean squared error (RMSE), across 19 cell types in the PBMC scMultiome dataset. DeepCNet achieves the best overall performance, with the highest median correlation (R = 0.745) and the lowest median RMSE (1.185). Among the competing methods, seq2cells-cell and seq2cells-celltype showed comparable correlation performance but substantially higher RMSE values, indicating reduced accuracy in capturing gene expression magnitudes. In contrast, Enformer and EPInformer showed lower correlations than seq2cells but relatively better RMSE performance. In the single-fold comparison with Enformer and Borzoi, DeepCNet again achieves highest median correlation (R = 0.777) and the lowest median RMSE (1.155) (Figure 2B). **Supplementary Figure S1A** illustrates observed versus predicted gene expression in regulatory T cells (Tregs), a rare cell type in the PBMC scMultiome dataset. We specifically compared DeepCNet with seq2cells-celltype, which is designed for cell type-specific gene expression from single cell data. DeepCNet predictions showed strong agreement with observed expression levels (R = 0.73) than seq2cells-celltype (R = 0.68), with predictions more closely distributed along the diagonal. Consistent with this pattern, DeepCNet achieved a substantially lower RMSE (1.125 vs. 2.399), indicating improved accuracy in capturing expression magnitude. Both methods showed greater prediction errors for lowly expressed genes, highlighting the increased difficulty of predicting genes in the low-expression range.

We further evaluated model performance on the MFC scMultiome dataset. Consistent with the PBMC results, DeepCNet demonstrated the strongest overall performance across eight major cell types, with most cell types achieving correlations above 0.7 and RMSE values below 1.5. DeepCNet attains the highest correlation in six out of eight cell types and the lowest RMSE across all cell types except pericytes (Per) and endothelial cells (End), both of which had relatively low cell abundance (Figure 2C**, Supplementary Table S1**). In the single-fold comparison with Enformer and Borzoi, DeepCNet again achieved higher median correlation (R = 0.752) and lower median RMSE (1.338) (Figure 2D). **Supplementary Figure S1B** further compares DeepCNet with seq2cells-celltype for microglia, a cell type strongly implicated in Alzheimer’s disease [10, 11]. DeepCNet showed stronger agreement with observed expression levels than seq2cells-celltype (R = 0.700 vs. 0.660), with predictions more closely distributed along the diagonal. Consistent with this pattern, DeepCNet achieved a substantially lower RMSE (1.267 vs. 2.161), indicating improved accuracy in capturing expression magnitude.

To assess the contribution of individual model components for gene expression prediction, we performed systematic ablation analyses (Methods). Removing the sequence-derived ExPecto embeddings had the largest impact in both datasets, followed by removing the cross-cell-type gene embeddings, which substantially reduced prediction performance in PBMC scMultiome dataset. Removing ATAC activity or peak-to-TSS distance resulted in comparatively modest reductions in prediction accuracy (**Supplementary Figure S2**).

Together, these results demonstrate that DeepCNet more accurately predicts cell type–specific gene expression than competing models, benefiting from integrating multimodal information. This improved predictive performance provides a strong foundation for downstream inference of promoter–enhancer interactions, consistent with prior evidence that explicitly modeling PE interactions can substantially enhance gene expression prediction accuracy [9].

### DeepCNet improves the prediction of cell type-specific promoter-enhancer interactions

We further benchmarked DeepCNet against competing methods for inferring PE interactions in the PBMC scMultiome dataset. As an orthogonal reference, we used cell type-specific ChIA-PET chromatin interactions from ENCODE (FDR < 0.1), covering six cell types matched to the PBMC scMultiome dataset (**Supplementary Table S2, S3**). PE interaction predictions were evaluated against the ChIA-PET-derived labels using the area under the receiver operating characteristic curve (AUROC) and area under the precision–recall curve (AUPRC) (Methods). Figure 3A summarizes the distribution of AUROC and AUPRC across evaluated genes, with median AUROC (mAUROC) reported for method comparison. Consequently, DeepCNet consistently ranked first across all six immune cell types, achieving the highest mAUROC, ranging from 0.658 to 0.680. EPInformer ranks second in five out of six cell types, with mAUROC values, ranging from 0.609 to 0.650. An unpaired Wilcoxon rank-sum test showed that DeepCNet significantly higher AUROC than EPInformer in five of six cell types (p < 0.05). Notably, both deep learning approaches outperform supervised approach, including DIRECT-NET (mAUROC range = 0.526–0.626) and SCARlink (mAUROC range = 0.545–0.603), as well as unsupervised methods, including ArchR (mAUROC range = 0.548–0.583) and Cicero (mAUROC range = 0.556–0.571). Analysis based on AUPRC shows a generally consistent pattern, with DeepCNet achieving the highest mAUPRC in most cell types (**Supplementary Figure S3A**).

We next evaluated method performance on the MFC scMultiome dataset using cell type-specific chromatin interactions derived from sn-m3C-seq (single-nucleus methyl-3C sequencing) data as an orthogonal reference [12]. Alignment between the MFC scMultiome and sn-m3C-seq datasets resulted in six matched cell types, which include Astrocytes (Ast), Endothelial cells (End), Excitatory neuron (Exc), Microglia (Mic), Oligodendrocyte (Oli) and Oligodendrocyte progenitor cells (Opc) (**Supplementary Table S1, S4**). Predicted cell type-specific PE interactions were evaluated against the sn-m3C-seq-derived chromatin interaction labels using gene-level AUROC and AUPRC (Methods). As shown in Figure 3B, DeepCNet showed substantially higher AUROC than competing approaches, achieving mAUROC values exceeding 0.80 across all six cell types. In comparison, EPInformer, DIRECT-NET, Cicero, ArchR, and SCARlink, achieved mAUROC ranges of 0.689–0.861, 0.488–0.580, 0.544–0.572, 0.490–0.575, and 0.539–0.591, respectively. A similar patten was observed for AUPRC, with DeepCNet outperforming competing approaches across all six cell types (**Supplementary Figure S3B**).

Moreover, we repeated the evaluation using a distance-matching strategy, in which positive and negative PE interactions were matched on TSS-to-peak distance (Methods). DeepCNet remained the best-performing method (**Supplementary Figure S4**). We further evaluated inferred PE interactions using independent CRISPR perturbation experiments (Methods). For PBMC scMultiome dataset, DeepCNet achieved mean AUROC values of 0.682 and 0.656, and mean AUPRC values of 0.362 and 0.358 for CD14 monocytes and CD16 monocytes, respectively. For MFC scMultiome dataset, DeepCNet achieved a mean AUROC value of 0.614 and a mean AUPRC value of 0.118 for Astrocytes. DeepCNet showed the strongest overall performance, with EPInformer ranking second in CD14 monocytes and Astrocytes. In CD16 monocytes, DeepCNet showed performance comparable to EPInformer, which achieved a mean AUROC of 0.653 and mean AUPRC of 0.350 respectively (**Supplementary Figure S5**). To assess the contribution of individual model components for PE interaction prediction, we performed systematic ablation analyses (Methods). Peak-to-TSS distance had the largest impact on performance, while removing gene embeddings also consistently reduced AUROC and AUPRC, particularly in the MFC scMultiome dataset. Removing ExPecto-derived embeddings or ATAC activity primarily affected AUROC (**Supplementary Figure S6**). Together, these results demonstrate that DeepCNet provides robust performance for inferring cell type-specific PE interactions across blood and brain tissues, with predictions that show strong concordance with multiple orthogonal reference datasets.

### DeepCNet enables the construction of cell type–specific TF–gene regulatory networks

DeepCNet enables the construction of cell type–specific TF-GRNs by integrating inferred PE interactions, TF binding potential at gene-linked ATAC peaks and cell type–specific TF expression (Methods). To illustrate this capability, we focused on three representative immune cell types, including CD4 central memory T (CD4 TCM) cells, natural killer cells (NK), and CD16 monocytes (CD16 Mono).

Figure 4A shows the top 20 TFs ranked by node strength in the TF-GRNs of these three cell types. Among these, 11 TFs were shared across all three cell types, including KLF2, KLF6, NFKB1, and RBPJ, highlighting common regulatory programs across immune cell types. KLF2 and KLF6 belong to the KLF family, which has well-known roles in immunity regulation [13, 14]. In particular, KLF2 plays a key role in regulating lymphocyte quiescence, trafficking, differentiation and function [15, 16]. NFKB1 encodes a core subunit of NF-κB and participates in NF-κB signaling involved in both innate and adaptive immune responses (Figure 4D) [17–19]. RBPJ, a key mediator of the Notch signaling, is essential for T cell and NK cell development and monocyte subset homeostasis [20–22]. In addition to shared regulators, DeepCNet identified TFs highlighting cell type–specific regulatory programs. In CD4 TCM cells, highly ranked TFs included MAZ, LEF1, CTCF, RFX3, and FOXP1. FOXP1 is a critical regulator of quiescence in CD4^+^ T cells [23–25], while LEF1 directs CD4+ T cell lineage decisions and drives a central memory program of natural killer T Cells [26, 27]. In CD16 monocytes, highly ranked TFs include KLF4, BACH1, TCF7L2 (TCF4), ZBTB7A, STAT1, REL, JUNB and SPI1 (PU.1). Several of these TFs have established roles in monocyte and myeloid biology, including KLF4, ZBTB7A, STAT1, JUNB, and SPI1 in monocytic or myeloid differentiation and maturation [28–35]. TCF7L2 as a transcriptional marker of immature CD16^+^ monocyte-derived dendritic cells, and REL and JUNB in inflammatory signaling and macrophage activation [36–38]. Together, these results demonstrate that the inferred TF-GRNs recover both shared and cell type-specific regulators consistent with established immune biology.

As a representative TF hub, we examined IRF1 (Interferon Regulatory Factor 1), one of the top-ranked TFs in CD4 TCM cells. IRF1 has established roles in T cell differentiation and activation, initiation of immune responses to pathogens, and the induction of interferons and pro-inflammatory cytokines [39–42]. In the inferred TF-GRN, IRF1 emerged as a high-connected hub with regulatory links to multiple target genes (Figure 4C). Gene Ontology (GO) enrichment analysis of top 1000 IRF1 targeted genes showed enrichment for biological processes related to T cell differentiation and activation, cytokine-mediated signaling and viral defense processes, consistent with the established functions of IRF1 (Figure 4B). Beyond individual TF hubs, DeepCNet also identified directed TF–TF relationships within the inferred TF-GRNs. As an illustrative example, we examined KLF2 and NFKB1 regulatory hubs in CD4 TCM cells (Figure 4E). GO enrichment analysis of target genes from the top 1,500 KLF2 and NFKB1 regulatory edges identified biological processes consistent with their established roles in immune regulation (Figure 4D). Notably, the inferred TF–TF relationship was directed from KLF2 to NFKB1, consistent with previous evidence that KLF2 modulates NF-κB-mediated transcriptional activity [43].

### DeepCNet-D enables the identification of differential promoter-enhancer interactions

We further evaluated DeepCNet-D, an extension of DeepCNet-S, designed to jointly model scMultiome data from two biological conditions and infer cell type-specific differential PE interactions between conditions (i.e., disease and healthy control). DeepCNet-D integrates shared peak sequences with condition-specific ATAC-derived chromatin accessibility from both conditions and simultaneously predicts gene expression for both conditions in a multi-task learning framework. Through its MHA-based chromatin interaction module, DeepCNet-D directly models differences in PE attention scores between the two conditions, denoted as $\Delta {\text{A}}_{\text{PE}}$ (DeepCNet-D) (Methods). To evaluate the advantage of joint modeling, we compared DeepCNet-D with a naïve two-model strategy, termed 2DeepCNet-S. In 2DeepCNet-S, two DeepCNet-S models are trained independently, one for each condition, and differential PE attention scores are obtained by subtracting the PE attention scores generated by the two models, denoted as $\Delta {\text{A}}_{\text{PE}}$ (2DeepCNet-S). As an illustrative application, we analyzed scMultiome data from one AD donor and one healthy control donor from the human medial frontal cortex (MFC) [44].

We first examined the distributions of difference of PE attention scores generated by DeepCNet-D and 2DeepCNet-S. The distributions of top 20% $\Delta {\text{A}}_{\text{PE}}$ (DeepCNet-D) and as $\Delta {\text{A}}_{\text{PE}}$ (2DeepCNet-S) show marked differences (Figure 5A–C, **Supplementary Figure S7**). For three major cell types: Exc, Mic and Oli, both scores exhibited right-skewed distributions, indicating large differences were concentrated in a relatively small proportion of PE interactions. Across all cell types, $\Delta {\text{A}}_{\text{PE}}$ (DeepCNet-D) was generally smaller, concentrated around 0.01–0.02, whereas $\Delta {\text{A}}_{\text{PE}}$ (2DeepCNet-S) was approximately two-fold larger, typically ranging from 0.02 to 0.05. This observation is consistent with the distinct modeling strategies of the two approaches. $\Delta {\text{A}}_{\text{PE}}$ (2DeepCNet-S) is derived from two independently trained models with separately estimated model parameters, including MHA parameters used to infer PE attention scores. Consequently, $\Delta {\text{A}}_{\text{PE}}$ (2DeepCNet-S) may reflect both condition-specific differences and variation arising from independently trained models. In contrast, $\Delta {\text{A}}_{\text{PE}}$ (DeepCNet-D) is derived from a single joint model in which most parameters are shared across conditions, while differential PE attention scores are captured through condition-specific MHA parameters. Therefore, DeepCNet-D is designed to more directly capture condition-specific differences while reducing variation arising from independently estimated model parameters.

To further evaluate this difference, we used a two-condition Hi-C data from an AD study (AD versus healthy control) as an orthogonal reference to compare cell type-specific differential PE interactions inferred by DeepCNet-D and 2DeepCNet-S (Methods) [45]. For each cell type, differential PE interactions were performed using the same distance-matching procedure and were evaluated using true discovery rate (TDR), defined as the proportion of Hi-C labelled differential PE interactions among PE pairs ranked by the absolute differential attention score $\left|\Delta {\text{A}}_{\text{PE}}\right|$ (**Supplementary Figure S8**). TDR was evaluated at increasing percentile thresholds (top 20%, 40%, 60%, 80%, and 100%) for both DeepCNet-D and 2DeepCNet-S. Enrichment trends across percentile thresholds were assessed using a one-sided Cochran–Armitage trend test implemented in the DescTools package. Across all six evaluated cell types, differential PE interactions inferred by DeepCNet-D generally achieved higher TDRs than those inferred by 2DeepCNet-S (Figure 5D–F**, Supplementary Figure S9**). Moreover, DeepCNet-D showed a statistically significant increasing enrichment trend, with higher TDRs among more highly ranked PE pairs, in three cell types, including excitatory neurons (Exc), microglia (Mic), and oligodendrocytes (Oli) (p < 0.05), whereas this pattern was absent or weaker for 2DeepCNet-S. These results support the advantage of jointly modeling two-condition scMultiome data with DeepCNet-D over independently fitting single-condition models for identifying differential PE interactions, as evidenced by greater concordance with differential chromatin interactions derived from the two-condition Hi-C data.

To further illustrate the interpretability of the differential attention scores, we examined two genes, CLU and SLC20A2, which have emerging roles in AD pathogenesis. CLU has been implicated in multiple AD-related biological processes, including amyloid-β and tau metabolism, lipid transport, immune modulation, amyloidogenesis, and microglia-dependent regulation of synaptic density [46–48]. Given the central role of microglia in AD-related neuroinflammation and adaptive immune responses, we therefore focused our subsequent analyses on microglia [10, 11]. For CLU, we identified a Hi-C loop present in the AD sample but absent in the healthy control, with one anchor located at the CLU promoter and the distal anchor overlapping a cluster of seven ATAC peaks (Figure 5G). DeepCNet-D assigned consistently positive $\Delta {\text{A}}_{\text{PE}}$ values to ATAC peaks within this cluster, with a clear local maximum relative to neighboring peaks. This pattern indicates that the direction and magnitude of the inferred differential PE attention scores are concordant with the condition-specific Hi-C interaction. In contrast, $\Delta {\text{A}}_{\text{PE}}$ values inferred by 2DeepCNet-S did not show a corresponding local maximum at the same peaks. We observed a complementary pattern for SLC20A2, which is highly expressed in the central nervous system and has been implicated in neurodegenerative diseases, including AD [49, 50]. Two Hi-C loops present in the healthy control but absent in AD sample were identified, each with one anchor at the SLC20A2 promoter and the other overlapping two clusters of ATAC peaks (Figure 5H). DeepCNet-D consistently assigned negative $\Delta {\text{A}}_{\text{PE}}$ values to peak within these clusters, concordant with the loss of the corresponding chromatin interactions in AD. Together, these results demonstrate that DeepCNet-D not only improves the enrichment of identified differential PE interactions against an orthogonal Hi-C reference but also provides interpretable differential attention scores that are concordant with condition-specific chromatin interactions, supporting the benefit of jointly modeling two-condition scMultiome data.

### DeepCNet reveals condition-specific change of TF-gene regulatory network

We next applied DeepCNet-D to two-condition scMultiome data from one AD and one healthy control, focusing on microglia, to characterize condition-specific change in TF-GRNs. DeepCNet-D infers differential PE interactions and constructs TF–peak–gene triplets, where peak-gene linkages are quantified by the absolute differential attention score $\left|\Delta {\text{A}}_{\text{PE}}\right|$. Differential TF-gene edge weights are then computed by aggregating triplet scores in a direction-aware manner, yielding cell type-specific differential TF-GRNs (Methods). Using this framework, we identify SPI1 (PU.1) as one of the top-ranked TFs exhibiting differential regulatory activity. SPI1 is a master regulator of microglial development and identity, and plays a central role in regulating inflammatory response in microglia and macrophage [34, 51]. It is also functions at multiple stages of myeloid differentiation [31–34, 52]. Importantly, SPI1 has been implicated as AD genetic risk and in the regulation of AD-associated inflammatory and amyloid-related processes [53–55]. We perform GO enrichment analysis on the top 1,000 SPI1-targeted genes ranked by differential TF–gene edge weights (Figure 6A). These genes were significantly enriched for biological processes related to myeloid cell differentiation and development, and immune and inflammatory response consistent with established functions of SPI1. Notably, enrichment was also observed for amyloid fibril formation, a process relevant to AD pathology [56]. In addition, several enriched processes were related to neuronal structure and synaptic function, including neuron projection extension, synaptic vesicle transport, and synaptic vesicle localization, which are relevant to neuronal connectivity and signaling affected in AD [57, 58].

To further illustrate differential TF–GRN structure, we visualized a SPI1-centered regulatory module consisting of the top 30 target genes, with edges indicating the direction of regulatory change between AD and control (Figure 6B). In microglia, SPI1-gene interactions involving targets such as AIF1, APOE, and PTK2B showed increased regulatory strength in the AD sample, whereas interactions with PKN1, PFDN5, and CDK5 exhibited decreased regulatory strength relative to the control. Several of these regulatory relationships are consistent with prior studies. For example, SPI1 has been reported as an up-regulator of AIF1 microglial expression in the human AD brain [59, 60], while AIF1 is deemed a pan-microglia marker [61]. We also observed increased SPI1-APOE regulatory strength in the AD sample, consistent with the evidence linking SPI1 to the regulation of multiple AD risk genes, including APOE, a major genetic risk factor for AD [51, 62]. Interestingly, APOEε4 has been shown to upregulate SPI1 expression, further implicating SPI1 in the APOE-microglia regulatory axis relevant to AD pathogenesis [63, 64].

Finally, SPI1-AIF1 regulatory linkage provides as an illustrative example of how differential attention scores can characterize condition-associated regulatory changes (Figure 6C). An ATAC peak upstream of the AIF1 promoter (chr6:31610935–31611435) showed a condition-specific difference in its inferred interaction with the AIF1 promoter. The differential PE attention score had a mean of 0.020 (SD = 0.013) across 50 independent DeepCNet-D runs, substantially higher than that of the second-ranked neighboring peak (mean = 0.006, SD = 0.009). In addition, AIF1 expression was significantly higher in the AD sample than in the healthy control (p = 1.83 × 10^−5^). The peak region also contained a SPI1 motif (P=0.0230; Methods), providing sequence-based evidence for potential SPI1 binding at this regulatory region. Together, these observations support the inferred condition-associated SPI1–AIF1 regulatory linkage.

## Discussion

In this study, we developed DeepCNet, a unified deep learning framework that integrates DNA sequence information with chromatin accessibility and cell type relationships derived from single-cell multiome data to jointly model gene expression and regulatory interactions. DeepCNet introduces several methodological advances that collectively enhance predictive and regulatory inference. First, DeepCNet leverages a pretrained sequence-to-function model to extract rich cell type-aware epigenomic features from promoter and ATAC peak sequences, facilitating efficient model training. Second, it incorporates a cross–cell-type autoencoder that learns joint gene embeddings from promoter accessibility, enabling information sharing across related cell types while preserving cell type-specific gene expression patterns. Third, by focusing explicitly on cis-regulatory regions represented by ATAC peaks, DeepCNet reduces model complexity and facilitates interpretability by restricting regulatory inference to candidate regulatory elements. Importantly, DeepCNet can be extended to two-condition scMultiome data by introducing learnable condition-specific parameters within the multi-head attention mechanism. This design enables the model to infer condition-dependent changes in promoter-enhancer interactions while maintaining a shared regulatory architecture across conditions. Together, these methodological advances enable DeepCNet not only to improve cell type-specific gene expression prediction but also to characterize how gene regulatory programs are organized across cell types and altered between biological conditions.

Benchmarking on two representative scMultiome datasets from blood and brain tissues demonstrated that DeepCNet consistently outperformed state-of-the-art approaches, including seq2cells, Enformer, EPInformer and Borzoi, in cell type-specific gene expression prediction, achieving higher correlations and lower root mean squared errors. These results indicate that DeepCNet more accurately captures both the relative variations and expression magnitudes. DeepCNet also outperformed methods such as DIRECT-NET, EPInformer, and SCARlink for PE interaction inference, as evaluated by AUROC and AUPRC against cell type-matched chromatin interaction and CRISPR perturbation data, and showed superior performance compared with ATAC-based methods, including ArchR and Cicero. Building upon the inferred PE interactions, DeepCNet further enables the construction of cell type–specific TF-GRNs. In the PBMC scMultiome dataset, DeepCNet identified shared and cell type-specific immune regulators, TF hubs and TF-TF regulatory relationships. Gene Ontology analyses showed that genes regulated by representative TF hubs were enriched for biological processes related to T-cell differentiation, immune responses, and signaling pathways, consistent with their established biological functions. As an illustrative two-condition application, we applied DeepCNet-D to scMultiome data from one AD donor and one healthy control donor. DeepCNet-D showed enrichment of inferred differential PE interactions against an orthogonal two-condition Hi-C reference and generated differential attention scores concordant with condition-specific chromatin interactions at AD-relevant loci. The analysis further highlighted candidate condition-associated regulatory alterations, including a SPI1-centered TF regulatory program in microglia. Given the two-donor design, these findings should be interpreted as illustrative candidate regulatory changes rather than population-level AD-associated effects.

Despite its strong performance, DeepCNet has several limitations that motivate future methodological development. First, because DeepCNet derives sequence embeddings from a pretrained sequence-to-function model, the learned sequence representations are less directly interpretable in terms of specific regulatory sequence features, such as TF binding motifs. Future work could incorporate one-hot-encoded DNA sequences more directly or develop sequence attribution approaches to improve sequence-level interpretability while maintaining predictive performance. Second, the current framework performs gene expression prediction and regulatory inference at cell type level. Aggregation reduces single-cell sparsity and facilitates robust modeling but inevitably obscures regulatory heterogeneity among individual cells. Extending DeepCNet to single-cell or finer cell-state resolution could enable direct characterization of within-cell-type regulatory heterogeneity and rare cellular states. Third, although the cross-cell type gene embeddings facilitate information sharing across related cell types, future extensions could explicitly account for differences in cell abundance during autoencoder training to improve representation learning for rare cell populations. In addition, the current implementation uses the reference genome as the sequence input. Incorporating individual-specific genomic sequences could enable DeepCNet to model the effects of genetic variation on gene expression and regulatory interactions.

Functional perturbation data provides another important opportunity for extending DeepCNet. Supervised learning based on experimentally characterized regulatory interactions is emerging as a complementary paradigm for PE interaction inference. For example, scE2G trains a logistic regression classifier using CRISPR-validated PE interactions in K562 together with multiome-derived features [65]. As tissue- and cell type-specific CRISPR perturbation datasets become increasingly available, DeepCNet could incorporate such labeled interactions during model training. Integrating functional perturbation data could improve the causal interpretation and predictive accuracy of inferred PE interactions. Finally, DeepCNet attention scores provide computational evidence for candidate PE interactions rather than direct mechanistic evidence of gene regulation. Validation remains constrained by the resolution, coverage, and availability of experimentally measured chromatin interaction and perturbation datasets in matched cell types and biological conditions. This limitation is particularly important for differential regulatory inference, where disease- and condition-specific functional perturbation datasets remain scarce. Accordingly, differential PE interactions identified by DeepCNet-D should be regarded as candidate regulatory changes rather than definitive causal relationships. Continued advances in single-cell chromatin conformation assays and condition-specific perturbation experiments will provide important opportunities for more comprehensive validation of DeepCNet-inferred regulatory mechanisms.

## Methods

### Single cell multiome data

We obtained the PBMC scMultiome dataset, in which RNA expression and chromatin accessibility were jointly profiled in the same cells, from 10x Genomics website and processed the data following the weighted nearest neighbor (WNN) workflow outlined in the Seurat vignette[66]. Gene expression and chromatin accessibility matrices were extracted and incorporated as separate modalities into a multimodal Seurat object, with ATAC modality processed using Signac[67]. Standard quality control filtering was applied, followed by SCTransform normalization for RNA expression and term frequency–inverse document frequency (TF–IDF) transformation with latent semantic indexing (LSI) for chromatin accessibility. A WNN graph was constructed by jointly leveraging RNA principal components and ATAC LSI embeddings, and cell clustering was performed based on the resulting graph. We retained only protein-coding genes based on the Homo sapiens Ensembl Release 86 annotation (EnsDb.Hsapiens.v86) and restricted analyses to genes located on chromosomes 1–22 and X. Cells expressing fewer than 600 genes were excluded. After quality control and filtering, the final PBMC dataset contained 10,532 cells profiled across 14,796 genes, spanning 19 annotated cell types. Detailed cell type relationships, cell type composition and sample sizes are provided in **Supplementary Figure S10 and Table S2**.

For the MFC scMultiome dataset, we used processed data with pre-annotated cell types from a human medial frontal cortex (MFC) study that profiled AD patients and healthy controls [44]. For gene expression prediction and PE interaction inference, we selected one healthy control sample (230211Kel_3, referred to as MFC_Normal). We additionally included one AD sample (230211Kel_19, referred to as MFC_AD) for differential PE interaction analyses. The same gene filtering strategy used for the PBMC dataset was applied, retaining only protein-coding genes on chromosomes 1–22 and X. Cells expressing fewer than 300 genes were excluded. After filtering, the healthy control sample and AD sample contained 6,464 cells and 5,153 cells, respectively, profiled across 17,743 genes. Cells were pre-annotated into eight major MFC cell types, with cell type relationships composition and sample size summarized in **Supplementary Figure S11 and Table S1**. For both the PBMC and MFC datasets, RNA expression and chromatin accessibility were aggregated across cells within each cell type, followed by log-transformation. The resulting cell type-level RNA expression values were used as model outputs, whereas cell type-level ATAC accessibility values were used as model inputs. All analyses of the PBMC and MFC scMultiome datasets were performed using the hg38 human reference genome.

### Chromatin interaction data

We collected chromatin interaction datasets to evaluate both cell type-specific and differential PE interactions. To evaluate cell type-specific PE interactions in the PBMC scMultiome dataset, we leveraged cell type–specific ChIA-PET datasets from ENCODE, selecting six cell types that could be matched in the PBMC scMultiome dataset (**Supplementary Table S2, S3**). For the MFC scMultiome dataset, we used the chromatin contact maps generated by sn-m3C-seq [12], which jointly profiles chromatin conformation and DNA methylation in the same nucleus. Six cell type–specific contact matrices were obtained to match the corresponding cell types in the MFC scMultiome dataset (**Supplementary Table S1, S4**). Chromatin interaction loops were identified from these contact matrices using FitHiChIP at a 1 kb resolution with an FDR cutoff of 0.1[68]. To ensure consistency with the scMultiome datasets, both anchors of each loop were lifted over from the hg19 to the hg38 reference genome. For both PBMC and MFC scMultiome datasets, an inferred PE interaction was labeled positive if the promoter overlapped one anchor of a chromatin interaction loop and the candidate enhancer overlapped the other anchor, and negative otherwise.

For evaluating cell type-specific differential PE interactions, we obtained Hi-C data from an AD study profiling from postmortem prefrontal cortex tissues, including four elderly individuals with AD and four similarly aged dementia-free individuals [45]. Chromatin interaction loops were called separately for each sample using HiCCUPS with an FDR cutoff of 0.1 [69]. To ensure sufficient loop coverage, we constructed condition-specific loop sets by taking the union of loops detected across samples within the AD and dementia-free groups, respectively. For a given a cell type, an inferred PE interaction was labelled as differential if it is overlapped a Hi-C loop detected exclusively in one condition and as non-differential if it overlapped loops in both conditions or in neither condition, allowing a 1 kb gap when determining overlap.

### CRISPR perturbation data

We used a combined CRISPR perturbation dataset generated in K562 cell line from ENCODE[3], which includes 10,412 gene-enhancer pairs (ENCSR998YDI; hereafter CRISPR-K562) to evaluate PE interactions inferred in CD14 monocytes and CD16 monocytes, two representative myeloid populations in the PBMC scMultiome dataset. For MFC scMultiome dataset, we used a CRISPRi dataset generated in human primary astrocytes from AstroREG, consisting of 7,759 gene-enhancer pairs (hereafter CRISPR-Ast), to evaluate PE interactions inferred in astrocytes [70]. Inferred PE interactions were matched to CRISPR-tested gene–enhancer pairs, allowing a positional gap of 1 kb when determining overlap. For the PBMC scMultiome dataset, matched PE interactions were labeled positive if the corresponding gene-enhancer pairs were designated “Significant” in CRISPR-K562 dataset and negative otherwise, resulting in 213 positive interactions among 1067 matched PE interactions. For the MFC scMultiome dataset, matched PE interactions were labeled positive if the corresponding gene-enhancer pairs had an FDR<0.1 in CRISPR-Ast dataset and negative otherwise, resulting in 94 positive interactions among 1379 matched PE interactions.

### Model architecture

#### Model architecture of DeepCNet-S

DeepCNet-S consists of 4 key components: (i) a sequence encoder (ii) a chromatin interaction module (iii) a cross-cell type encoder and (iv) a gene expression prediction module. Gene expression and chromatin accessibility were quantified at the cell type level by aggregating RNA-seq and ATAC-seq read counts across cells within each cell type, followed by log transformation. The sequence encoder transforms DNA sequences from gene promoters and nearby ATAC peaks (i.e. candidate enhancers) into tissue- and cell type-aware epigenomic features using the pretrained sequence-to-function model ExPecto [1]. The promoter region is defined as ±1 kb of TSS. For each gene, the promoter and its nearest n ATAC peaks within ±100 kb of the TSS are retained as model inputs (default n = 60 total peaks). All peak sequences are resized to a uniform length of 2-kb. For genes with fewer than 60 peaks, pseudo-peaks consisting of 2-kb “N”- padded DNA sequences are added to ensure consistent input dimensionality across genes. One-hot–encoded DNA sequences from the promoter and ATAC peaks are passed through the pretrained ExPecto model [1], yielding tissue- and cell type-aware epigenomic feature embeddings $X_{\text{epi}}\in R^{(n+1)\times 2002}$. These embeddings are projected to a lower-dimensional space using a feature projection module composed of two consecutive one-dimensional convolutional layers (kernel size = 1, output dimension = 64, 64), each followed by batch normalization and a ReLU activation. The resulting features $X_{\text{epi}}\in R^{(n+1)\times 64}$ are concatenated with ATAC-derived features $X_{\text{atac}}\in R^{(n+1)\times 2}$, which include log-transformed peak-to-TSS distance and chromatin accessibility for both promoter and ATAC peaks. The combined feature matrix $X_{\text{epi}+\text{atac}}\in R^{(n+1)\times 66}$ is subsequently passed through two feature fusion blocks, each consisting of a 1D convolutional layer (kernel size = 1, output dimension = 64) followed by a ReLU activation, producing the input to the chromatin interaction module, $X_{\text{MHA}}^{\text{in}}\in R^{(n+1)\times 64}$. The chromatin interaction module uses an MHA encoder to model PE interactions and generates both contextualized embeddings for the promoter and candidate enhancers $X_{\text{MHA}}^{\text{out}}\in R^{(n+1)\times 64}$ and a PE attention vector $A_{\text{PE}}\in R^{1\times (n+1)}$ that quantifies the inferred interaction strength between the promoter and each ATAC peak (MHA Encoder). The first row of $X_{\text{MHA}}^{\text{out}}$, denoted $X_{\text{MHA}}^{\text{prom}}\in R^{1\times 64}$, represents contextualized promoter embedding learned by integrating sequence-derived features, chromatin accessibility, peak-to-TSS distance, and interactions with surrounding ATAC peaks. $X_{\text{MHA}}^{\text{prom}}$ is concatenated with a learnt gene embedding $X_{\text{enc}}^{\text{prom}}\in R^{1\times 32}$, generated by the cross-cell type autoencoder (*cross-cell type autoencoder*), to produce a joint gene embedding $X_{\text{joint}}^{\text{prom}}\in R^{1\times 96}$, which serves as input to gene expression prediction module. The prediction module consists of three fully connected layers with output dimensions (128, 128, 1). ReLU activations are applied between layers, and the final layer produces the predicted gene expression value Y.

#### MHA Encoder

The MHA encoder consists of three sequential and identically structured transformer encoder blocks. In each block, the input first undergoes layer normalization, followed by an eight-head self-attention mechanism with a total embedding dimension of 64 (8 dimensions per head). For self-attention, the query, key and value matrix are defined as $Q=K=V=X_{\text{MHA}}^{\text{in}}\in R^{(n+1)\times 64}$. Multi-head attention is computed as,

$$X_{\text{attn}}=\text{MultiHead}(Q,K,V)=\text{Concat}\left({\text{head}}_{1},{\text{head}}_{2},\ldots {\text{head}}_{8}\right)W^{O}$$

where

$${\text{head}}_{i}=\text{Attention}\left(QW_{i}^{Q},KW_{i}^{K},VW_{i}^{V}\right)$$

$W_{i}^{Q}\in R^{d_{0}\times d_{q}},W_{i}^{K}\in R^{d_{0}\times d_{k}},W_{i}^{V}\in R^{d_{0}\times d_{v}}$ *and* $W^{O}\in R^{hd_{v}\times d_{0}}$. Here, $d_{0}=64$, the number of heads h=8, and $d_{k}=d_{v}=d_{0}/h=64/8=8$.

Attention is computed as,

$$\text{Attention}(Q,K,V)=\text{softmax}\left(\frac{QK^{T}}{\sqrt{d^{k}}}+M\right)V$$

where $M\in R^{\left(n+1)\times (n+1\right)}$ denotes the attention mask matrix. All entries in M are set to negativity infinity except those in the first row corresponding to promoter-enhancer interactions, and padding positions are additionally masked. This design restricts attention to PE interactions while excluding promoter–promoter and enhancer–enhancer interactions. The PE interaction score between promoter and enhancer i, is computed as $A_{PE_{i}}=\text{softmax}\left(\frac{q^{T}K_{i}}{\sqrt{d^{k}}}\right)$, where q, first row of Q, denotes the promoter query vector and $K_{i}$, ith row of K, denotes the enhancer key vector. PE interaction scores are concatenated into an attention score vector as $A_{\text{PE}}=\left[A_{PE_{1}},A_{PE_{2}},\ldots ,A_{PE_{n}}\right]$. The final $A_{PE}$ is obtained by averaging the attention scores across all eight heads and three encoder blocks. The multi-head attention output $X_{\text{attn}}$ passes through a residual connection, a second layer normalization, and a feed-forward network composed of two fully connected layers (384,64) with a ReLU activation in between. Another residual combines the feed-forward output with its input. After three stacked encoder blocks, the final MHA output feature matrix $X_{\text{MHA}}^{\text{out}}\in R^{(n+1)\times 64}$ is forwarded to the gene expression prediction module.

#### Cross cell-type autoencoder

Cross cell-type autoencoder is designed to learn the cross-cell type gene embeddings by leveraging single-cell ATAC accessibility at gene promoters. For each gene, single-cell gene activity $X_{\text{act}}\in R^{1\times N}$ is calculated using the GeneActivity function from Signac [67], which quantifies ATAC accessibility within promoter regions for all cells within each cell type. Let $N_{c}$ denotes the number of cells for cell type c, and let N denotes the total number of cells across all C cell types, such that $N=\sum_{c=1}^{C}N_{c}$. The gene activity for cells belonging to cell type c is denoted as $X_{\text{act}}^{c}\in R^{1\times N_{c}}$. For each cell type c, a cell type-specific encoder takes $X_{\text{act}}^{c}$ as input and generates a cell type–specific gene embedding $X_{c}^{\text{prom}}\in R^{1\times 8}$. Each cell type-specific encoder consists of two fully connected layers with output dimensions of 64 and 8, respectively, with a ReLU activation layer between the two layers. The embeddings generated by all cell type-specific encoder are stacked and passed through a fusion block composed of a 1D convolutional layer (kernel size = 3, padding = 1), followed by a ReLU activation, dropout, and an adaptive average pooling layer to generate the final cross-cell type gene embedding $X_{\text{enc}}^{\text{prom}}\in R^{1\times 32}$. A cell type-agnostic decoder takes $X_{\text{enc}}^{\text{prom}}$ as input and reconstructs the original gene activity $X_{\text{act}}$. The decoder consists of two fully connected layers with output dimensions of 128 and N, connected by a ReLU activation. The cross cell-type autoencoder is trained independently from the main DeepCNet model. The total loss function is defined as $L_{\text{total}}=L_{\text{Recon}}+\lambda L_{\text{ct}}$, where λ controls the relative contribution of the regularization term. The reconstruction loss $L_{\text{Recon}}$ is computed as the mean squared error (MSE) between the original and reconstructed gene activities as $L_{\text{Recon}}=\text{MSE}\left(X_{\text{act}},\hat{X_{\text{act}}}\right)$. The cell type similarity loss $L_{\text{ct}}$ serves as a regularization term that encourages more similar gene embeddings between more closely related cell types. It is formulated as a weighted pairwise loss as $L_{\text{ct}}=\sum_{i,j}w_{\text{ij}}\times \text{MSE}\left(X_{i}^{\text{prom}},X_{j}^{\text{prom}}\right)$, where $w_{\text{ij}}$ quantifies the relationship between cell type i and j (Cell-type relationship characterization)

#### Cell-type relationship characterization

To characterize the cell type relationships, we define the similarity weight $w_{\text{ij}}$ using either prior biological knowledge or empirical estimation from chromatin accessibility, depending on data availability. When biological knowledge is available, such as cell lineage tree or cell ontology tree, the similarity between cell type i and j is quantified by applying an exponential decay function to their topological distances $d_{\text{ij}}$, defined as $w_{\text{ij}}=\text{exp}\left(-\alpha d_{\text{ij}}\right)$, where α>0 (default 0.5) controls the decay rate with increasing topological distance. Alternatively, cell type relationships can be estimated empirically from chromatin accessibility. In this setting, each cell type is represented by low-dimensional ATAC embeddings defined by the top 10 principal components of the chromatin accessibility data. The distance $d_{\text{ij}}$ between cell type i and j is then defined as the optimal transport (OT) distance with a Euclidean cost between their corresponding ATAC embeddings. Because computing all pairwise cell type distances $d_{\text{ij}}$ can be computationally expensive as the number of cell types increases, we adopt an anchor-based approximation in practice. Specifically, a single anchor cell type is selected based on cell type abundance and its topological centrality within the cell type relationship graph. The cell type similarity loss is then simplified to $L_{\text{ct}}=\sum_{i}w_{i,\text{anchor}}L_{\text{ct},i}=\sum_{i}w_{i,\text{anchor}}\times \text{MSE}\left(X_{i}^{\text{prom}},X_{\text{anchor}}^{\text{prom}}\right)$, where $X_{i}^{\text{prom}}$ and $X_{\text{anchor}}^{\text{prom}}$ denote the gene embeddings for cell type i and the anchor cell type, respectively. The cell-type relationships used to calculate the similarity weights for PBMC and MFC scMultiome datasets are shown in Supplementary **Figure S10** and **S11**, respectively. CD4 TCM cells were selected as the anchor cell type for PBMC dataset, whereas excitatory neurons were chosen as the anchor cell type for the MFC dataset.

#### DeepCNet-D

DeepCNet-D is developed to infer differential PE interactions between two biological conditions using a multi-task learning framework that extends DeepCNet-S to jointly model condition-specific input and output. Specifically, DeepCNet-D uses a common set of ATAC peaks jointly identified across all cells from both conditions. The sequence embedding, denoted as $X_{\text{epi}}$, generated from the sequence encoder, captures tissue- and cell type-aware epigenomic features for each promoter and ATAC peak. Because the same set of genomic regions is used across conditions, the sequence embeddings and peak-to-TSS distances are shared across conditions, whereas chromatin accessibility is condition-specific. The ATAC-derived features for the two conditions, denoted as $X_{\text{atac}}^{1}$ and $X_{\text{atac}}^{2}$, therefore contain the shared peak-to-TSS distance together with the corresponding condition-specific chromatin accessibility. The DeepCNet-D input consists of two feature sets as ($\left[X_{\text{epi}},X_{\text{atac}}^{1}\right],\left[X_{\text{epi}},X_{\text{atac}}^{2}\right]$), which are processed through parallel branches following the same feature projection, fusion and chromatin interaction procedures as DeepCNet-S. These steps generate condition-specific inputs to the chromatin interaction module, $X_{\text{MHA}}^{\text{in},1},X_{\text{MHA}}^{\text{in},2}\in R^{(n+1)\times 64}$, Rather than using two independent MHA encoders, DeepCNet-D employs a shared MHA encoder with learnable condition-specific parameters to generate condition-specific contextualized embeddings and PE interaction scores. This design enables the model to learn a shared regulatory patterns across conditions, while capturing condition-specific differences in PE interactions through additional parameters, thereby reducing model complexity. Specifically, each transformer encoder block of the shared MHA encoder incorporates a conditional-specific learnable parameter $b_{c}$ that modulates PE interactions,

$${\text{Attention}}_{c}(Q,K,V)=\text{softmax}\left(\frac{QK^{T}}{\sqrt{d^{k}}}+M+ob_{c}\right)V,c\in \{1,2\}$$

Here, $o\in R^{(n+1)\times 1}$ is a one-hot encoding vector corresponding to the first row of attention mask M, which represents PE interactions. The learnable parameter $b_{c}\in R^{1\times (n+1)}$ encodes condition-specific modulation of PE attention, while all other parameters follow the MHA encoder defined for DeepCNet-S. Consequently, the shared MHA encoder yields condition-specific outputs, including contextualized embeddings $X_{\text{MHA}}^{C}$ and PE interaction scores $A_{\text{PE}}^{C},c\in \{1,2\}$. The differential PE interaction is defined as

$$\Delta A_{PE}=A_{PE}^{1}-A_{PE}^{2}$$

The condition-specific embeddings $X_{\text{MHA}}^{C}$ are subsequently forwarded to the gene expression prediction module, which follows the same architecture as DeepCNet-S, except that the final output layer contains two output nodes corresponding to predicted gene expression levels for the two biological conditions.

### Model training and testing

We trained DeepCNet and the benchmark methods using 12-fold cross-chromosome validation, following the strategy implemented in EPInformer, to construct training, validation and testing sets. For folds 1–10, chromosomes i and i+10 were reserved for validation, whereas chromosomes i+1 and i+11 were used for testing (i=1,…,10). In fold 11, chromosomes 3 and 21 served as the validation set, and chromosomes 22 and X were held out for testing. In fold 12, chromosomes 2 and 22 were reserved for validation, with chromosome 1 designated as the test set. All remaining chromosomes were used for model training in each fold. For gene expression prediction, predictions from the held-out test genes across all 12 folds were concatenated to generate a genome-wide held-out prediction set for each cell type. For single-fold evaluations, performance metrics were calculated using only the test genes from the selected fold. For PE interaction inference, AUROC and AUPRC were calculated for each gene in each training fold and then averaged across all folds for that gene. For DeepCNet-S, the final output layer produces the predicted gene expression $\hat{Y}$, and the model is trained using the SmoothL1loss $L=\text{SmoothL}1\text{Loss}(Y,\overset{ˆ}{Y})$. For DeepCNet-D, gene expression is predicted simultaneously for two biological conditions, yielding ${\hat{Y}}_{1}$ and ${\hat{Y}}_{2}$. The training loss is defined as the average Smoothed L1 loss across the two conditions $L=\frac{1}{2}\left[\text{SmoothL}1\text{Loss}\left(Y_{1},{\hat{Y}}_{1}\right)+\text{SmoothL}1\text{Loss}\left(Y_{2},{\hat{Y}}_{2}\right)\right.$. Both DeepCNet-S and DeepCNet-D were trained using the AdamW optimizer with a learning rate of 5 × 10^−4^ and a weight decay of 1 × 10^−6^. Models were trained for a maximum of 30 epochs with early stopping using a patience of 5 epochs. For DeepCNet-S, early stopping was based on the validation coefficient of determination (R^2^) between observed and predicted gene expression. For DeepCNet-D, early stopping was based on the mean validation R^2^ across the two conditions. DeepCNet contain approximately 0.32 million trainable parameters.

### Benchmarking methods and performance evaluation

To benchmark cell type-specific gene expression prediction, we compared DeepCNet with five state-of-the-art models, including Enformer, EPInformer, seq2cells, ExPecto and Borzoi [1, 2, 7–9]. Enformer was originally designed to take ~196 kb DNA sequences as input to jointly predict 5,313 genomic tracks simultaneously. To adapt Enformer for cell type-specific gene expression prediction, we initialized the model using the pretrained EleutherAI/enformer-official-rough checkpoint and replace the original 5,313-track output heads with a newly initialized linear layer followed by a Softplus activation to produce a single-track output track. Following the Enformer protocol, gene-level expression was calculated by summing coverage across the 128-bp bin overlapping the TSS and its two neighboring bins. Both the pretrained Enformer trunk and the newly initialized output head were updated during model training. The model was optimized using Poisson loss and the Adam optimizer, with a learning rate of 5 × 10^−5^, a batch size of 2, and a maximum of 50 epochs. Early stopping was applied with a patience of 5 epochs based on the validation loss, and the checkpoint with the lowest validation loss was retained for evaluation. For EPInformer, we adopted EPInformer-PE-Activity, a transformer-based approach that predicts gene expression by integrating promoter and enhancer DNA sequences, promoter-enhancer distances, and enhancer activities. To adapt EPInformer to single-cell multiome setting, enhancers were defined as ATAC peaks and enhancer activities were quantified using ATAC read counts, consistent with the feature definitions used in DeepCNet. Seq2cells, which builds upon Enformer, uses a transfer-learning framework to predict gene expression at either the single-cell or cell type level from single-cell RNA-seq data. We evaluated both prediction modes, denoted as seq2cells-cell and seq2cells-celltype. Seq2cells-celltype directly predicts gene expression at the cell type level, whereas seq2cells-cell predicts expression at the single-cell level followed by aggregation to the cell type level. ExPecto was implemented following its default settings. It uses a deep convolutional neural network to predict 2,002 epigenomic features from DNA sequence across a 40-kb region centered on the TSS, followed by spatial transformation and a L2-regularized linear model to predict tissue- or cell type-specific gene expression. Borzoi was implemented using the Borzoi/Baskerville training framework, which used 524 kb DNA sequences to predict RNA-seq coverage for all cell types in a multitask setting. We used the “Mini Borzoi” configuration, containing approximately 42.5 million parameters, and trained the full model end-to-end from scratch. Training was performed using the Adam optimizer with a learning rate of 1 × 10^−4^, a batch size of 2, and Poisson multinomial loss. Models were trained for a minimum 60 and a maximum of 100 epochs, with an early stopping patience of 10 epochs based on validation performance. The best checkpoint was selected using the default validation criterion, which is defined as validation Pearson correlation plus one quarter of validation R^2^.

To evaluate cell type-specific PE interaction inference, we compared DeepCNet-S with four widely used approaches, including Cicero, ArchR, DIRECT-NET, SCARlink and additionally included EPInformer due to because its attention-based architecture enables PE interaction inference. Cicero identifies PE interactions using scATAC-seq data by estimating co-accessibility between peaks and gene promoters using graphical lasso on aggregated cell [71]. ArchR estimates peak co-accessibility by computing Pearson correlations between log2-normalized accessibility of peaks and gene promoters using pseudo-bulk cells [72]. DIRECT-NET models gene expression as a function of chromatin accessibility using XGBoost and uses feature importance scores to quantify regulatory associations [73]. SCARlink employs regularized Poisson regression to model gene expression as a function of chromatin accessibility, with the estimated coefficients used to quantify PE interactions. For all compared methods, high scores indicate stronger PE interactions. All approaches were implemented using default parameters as specified in their original publications. For each gene, AUROC and AUPRC were computed using the binary labels (Chromatin interaction data) and the corresponding inferred PE interaction scores. Analyses were restricted to genes with more than 10 peaks and sufficient positive and negative interactions, defined as at least five positive and five negative interactions for the PBMC scMultiome dataset and at least three positive and three negative interactions for the MFC scMultiome dataset. To account for differences in TSS-to-peak distance distributions between positive and negative PE interactions, we performed an additional distance-matching evaluation by using nearest-neighbor propensity score matching without replacement based on the log-transformed TSS-to-peak distance. Matched datasets were constructed at positive-to-negative ratio of 1:1 for PBMC scMultiome dataset and 1:5 for MFC scMultiome dataset.

For MFC scMultiome dataset, to benchmark cell type-specific differential PE interactions against bulk Hi-C data, we restricted the evaluation to cell type-specific marker genes, following the strategy adopted by DIRECT-NET to facilitate cell type-specific comparison. Marker genes were identified by differential expression analysis using Seurat’s FindAllMarkers function with min.pct=0.1 and logfc.threshold=0.1. For each cell type, differential PE interactions were evaluated using TDR, defined as the proportion of Hi-C labelled differential PE interactions (Chromatin interaction data) among the top-ranked PE pairs based on absolute differential attention score $\left|\Delta {\text{A}}_{\text{PE}}\right|$. We applied the same distance-matching procedure to positive and negative differential PE interactions, using a 1:5 positive-to-negative ratio. Per and End were excluded from this evaluation because relatively few differential PE interactions were identified for these cell types (**Supplementary Figure S8**). To assess the benefit of joint two-condition modeling with the shared MHA encoder in DeepCNet-D, we further compared DeepCNet-D with an alternative approach, termed 2DeepCNet-S, which independently fits a DeepCNet-S model to each condition and calculates differential PE attention scores as the difference between the attention scores generated by the two models.

### Ablation analyses

To evaluate the contribution of individual model components, we performed systematic ablation analyses by comparing the full DeepCNet model with four ablated variants: (1) w/o gene embedding, in which gene embeddings learned by the cross-cell-type autoencoder were removed; (2) w/o activity, in which ATAC-derived chromatin accessibility was excluded; (3) w/o distance, in which peak-to-TSS distance was excluded; and (4) w/zero sequence embedding, in which ExPecto-derived sequence embeddings were zero-masked.

### Cell type-specific TF-gene regulatory network

Using PE interactions inferred by DeepCNet-S, we constructed cell type-specific TF-GRNs through three main steps, following a recent developed framework with appropriate modifications [5]. First, we selected top PE interactions (i.e. 20%) ranked by PE attention score $A_{PE}$ for downstream motif analysis. TF motif scanning was performed using motifmatchr R package with position weight matrices (PWMs) from JASPAR2020 as the reference database [74]. For each TF-peak pair, motif scanning generated an observed motif score, denoted as $S_{\text{obs}}$. To account for sequence composition bias, the observed motif score was normalized using background peaks. Specifically, $n_{\text{bg}}$ background peaks $\left(n_{\text{bg}}=1000\right)$ were generated using chromVAR by matching GC content and peak length [75]. Motif scanning was then performed on each background peak j, yielding a background motif score $S_{\text{bg}}^{j}$. An empirical p-value was calculated as $p_{\text{empirical}}=1-\frac{\sum_{j=1}^{n_{\text{bg}}}I\left(S_{\text{obs}}\geq S_{\text{bg}}^{j}\right)+1}{n_{\text{bg}}+1}$ and subsequently transformed into a normalized motif score $TF_{\text{score}}=-{\text{log}}_{10}\left(p_{\text{empirical}}\right)$, representing the relative strength of TF motif enrichment at each peak. Second, TF–peak–gene triplets were constructed by integrating TF-peak and peak-gene associations. TF-peak pairs were identified by selecting peaks with significant TF motif enrichment ($p_{\text{empirical}}<0.05$). These TF-peaks pairs were further filtered based on TF expression, requiring an average log-normalized expression greater than 0.1 and detectable TF expression in more than 10% of cells in the corresponding cell type. Peak-gene associations were inferred from DeepCNet-S using the PE attention score $A_{PE}$. To ensure comparability across genes, $A_{PE}$ values were normalized on a per-gene basis by rescaling ranks of gene-linked peaks to the interval [0,1]. For each TF–peak–gene triplet, a triple score was defined as the geometric mean of three components, including normalized motif score $\left(TF_{\text{score}}\right)$, TF expression level $\left(TF_{\text{exp}}\right)$, and normalized PE attention score $\left(A_{PE}\right)$ as $S_{\text{Ttr}}={\left(TF_{\text{score}}\times TF_{\text{exp}}\times A_{PE}\right)}^{1/3}$. Finally, cell type-specific TF-GRN was constructed by aggregating multiple TF–peak–gene triplets, with the TF–gene edge weight defined as the sum of triple scores across all peaks linking a given TF to a target gene as $W_{\text{TF}-\text{gene}}=\sum_{\text{peak}}S_{\text{Ttr}}(\text{TF},\text{gene},\text{peak})$.

### Cell type-specific differential TF-gene regulatory network

Leveraging DeepCNet-D, we extended the TF-GRN framework to construct cell type-specific differential TF-GRNs between two biological conditions. We first selected top-ranked inferred differential PE interactions (e.g. 20%) based on the absolute differential attention score $\left|\Delta {\text{A}}_{\text{PE}}\right|$. To isolate regulatory changes driven by differential PE interactions rather than differences in TF abundance, TF expression from reference condition only (e.g., healthy control) was used for both conditions. This design reduces the contribution of differential TF expression to the inferred TF-GRN differences, thereby emphasizing changes associated with differential PE interactions. TF–peak–gene triplets were then constructed following the same procedure used for cell type–specific TF-GRN, with one key modification. The peak-gene scores for each triplet were defined using the absolute differential PE attention score $\left|\Delta {\text{A}}_{\text{PE}}\right|$. Differential TF–gene edge weight were computed by aggregating triplet scores across all peaks linking each TF to its target gene in a direction-aware manner, with the edge weight defined as $W_{TF-\text{gene}}=|\sum_{\text{peak}}S_{\text{Ttr}}(\text{TF},\text{gene},\text{peak})\times \text{sign}\left(\Delta {\text{A}}_{\text{PE}}\right)|$ and its direction as $D_{\text{TF}-\text{gene}}=\text{sign}(\sum_{\text{peak}}S_{\text{Ttr}}(\text{TF},\text{gene},\text{peak})\times \text{sign}(\Delta A_{PE}))$. A positive differential edge weight indicates inferred TF–gene regulatory strength in one condition relative to the other, whereas a negative value indicates decreased regulatory strength. Together, this formulation enables DeepCNet-D to characterize both the magnitude and direction of TF–gene regulatory changes associated with condition-specific alterations in PE interactions.

## Supplementary Files

This is a list of supplementary files associated with this preprint. Click to download.

- DeepCNetsuppl.0831.pdf
- nrreportingsummary.pdf
- 6984411supp1345402tkpvss.pdf

## Figures and Tables

**Figure 1.**
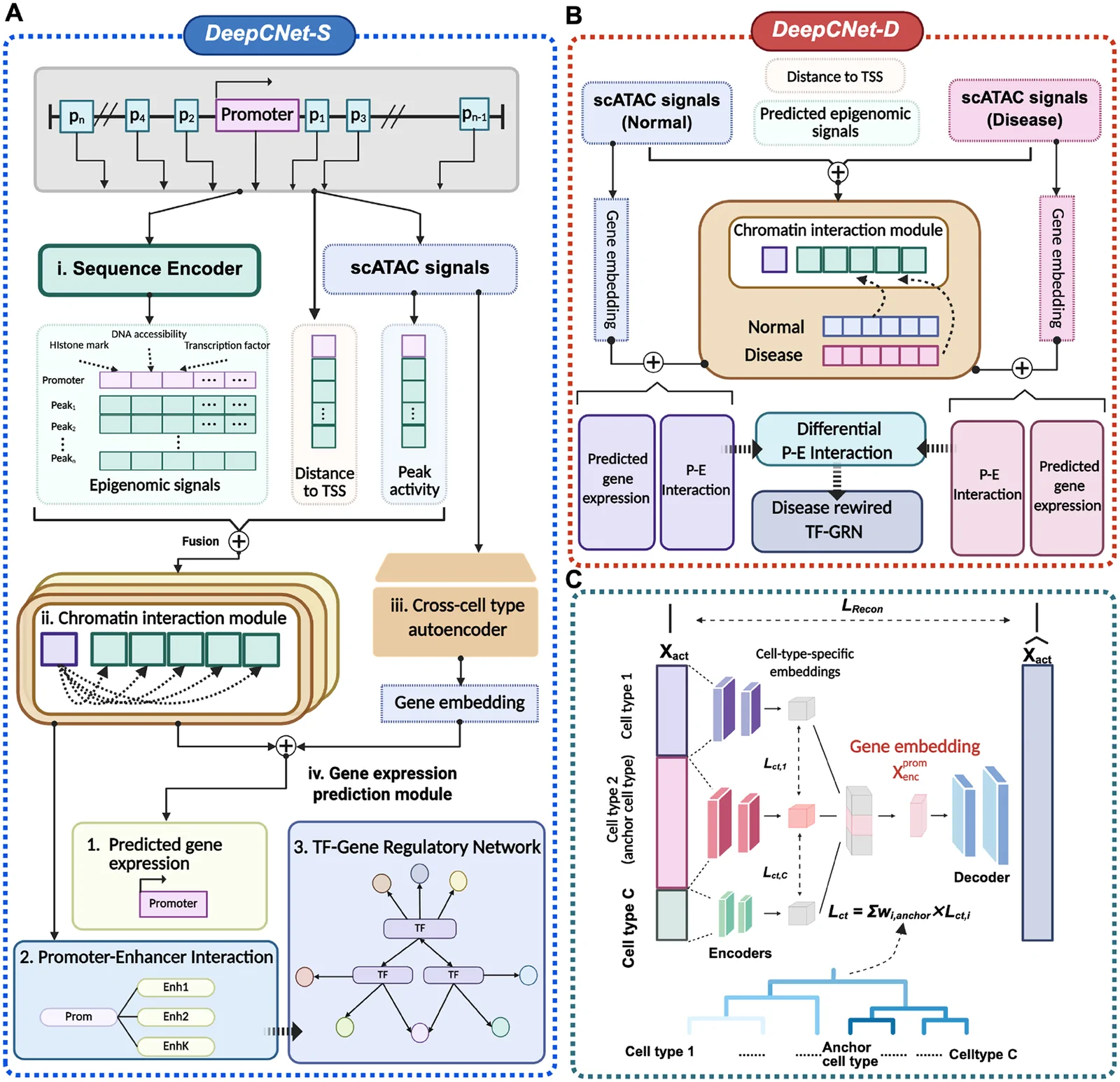
Overview of DeepCNet. **A. DeepCNet-S.** DeepCNet-S focuses on predicting cell type-specific gene expression, inferring PE interactions and constructing TF-GRNs. For each gene, one-hot encoded DNA sequence and chromatin accessbility of the promoter and nearby ATAC peaks within ±100 kb of TSS (default n=60) are used as model inputs. DNA sequences are first processed by the (i) sequence encoder to generate tissue and cell type–specific epigenomic features, including histone mark, DNA accessibility and transcription factor, which are then integrated with ATAC-derived features, including peak-to-TSS distance and chromatin accessibility. The resulting features are passed to (ii) chromatin interaction module, which models PE interactions using multi-head attention (MHA) encoder. In parallel, cell type-aware gene embeddings are learned from chromatin accessibility across cell types through the (iii) cross–cell type autoencoder. Finally, embeddings from the chromatin interaction module and the cross–cell type autoencoder are integrated by the (iv) gene expression prediction module to generate cell type–specific gene expression predictions. **B. DeepCNet-D.** DeepCNet-D extends DeepCNet-S to jointly model two biological conditions (e.g., disease vs. control) to identify differential PE interactions and characterize atlerations in TF-GRNs. For each gene, paired inputs from two conditions consist of shared DNA sequence from a common set of ATAC peaks and condition-specific chromatin accessibility. The paired features are processed by a shared MHA-based chromatin interaction module with learnable condition-aware parameters to generate condition-specific PE interaction scores. Gene embeddings for the two conditions are learned using separate cross–cell type autoencoders, and gene expression is predicted simultaneously for two conditions. Differences in inferred PE interactions between conditions are used to characterize differential PE interactions and associated alterations in TF-GRNs. **C. Cross-cell type autoencoder.** The autoencoder takes aggregated gene activity derived from single-cell ATAC-seq data as input and applies cell type–specific encoders to generate gene embeddings for each cell type. The cell type-specific gene embeddings are subsequently integrated into a joint gene embedding, from which a shared, cell type–agnostic decoder reconstructs the original gene activity. Model training is guided by a composite objective combining a reconstruction loss and a cell type similarity loss that promotes consistency among related cell types. An anchor cell type is selected based on cell type abundance and its topological position within the cell type relationship graph.

**Figure 2.**
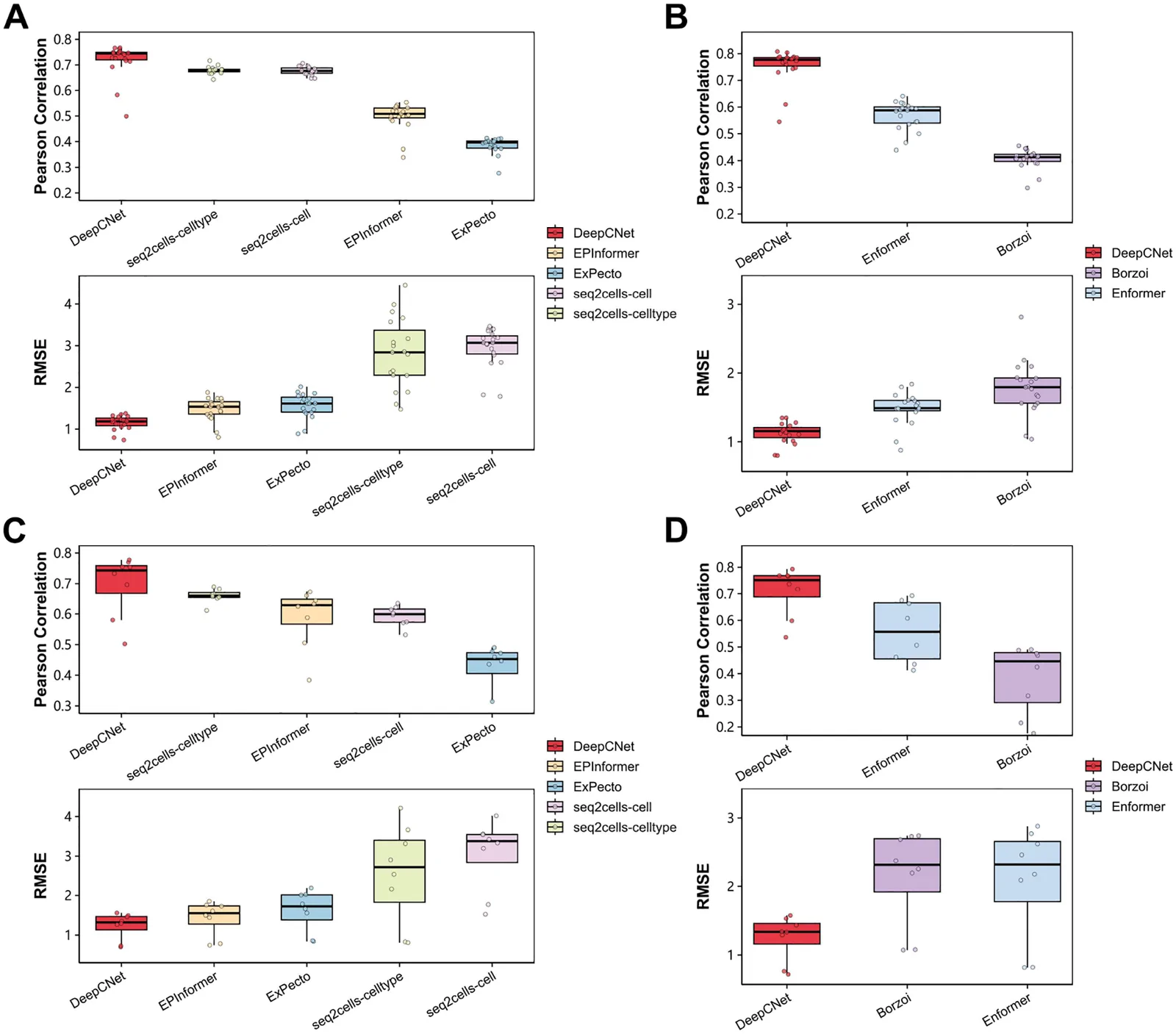
Benchmarking DeepCNet for gene expression prediction. Prediction performance was evaluated using Pearson correlation (R) and root mean squared error (RMSE). Each point in the boxplot represents one cell type, and methods are ordered by decreasing median R or increasing median RMSE. **A.** R and RMSE across all cell types on the PBMC scMultiome dataset using 12-fold cross-chromosome validation. **B.** R and RMSE across all cell types on the PBMC scMultiome dataset using single-fold cross-chromosome validation. **C.** R and RMSE across all cell types on the MFC scMultiome dataset using 12-fold cross-chromosome validation. **D.** R and RMSE across all cell types on the MFC scMultiome dataset using single-fold cross-chromosome validation.

**Figure 3.**
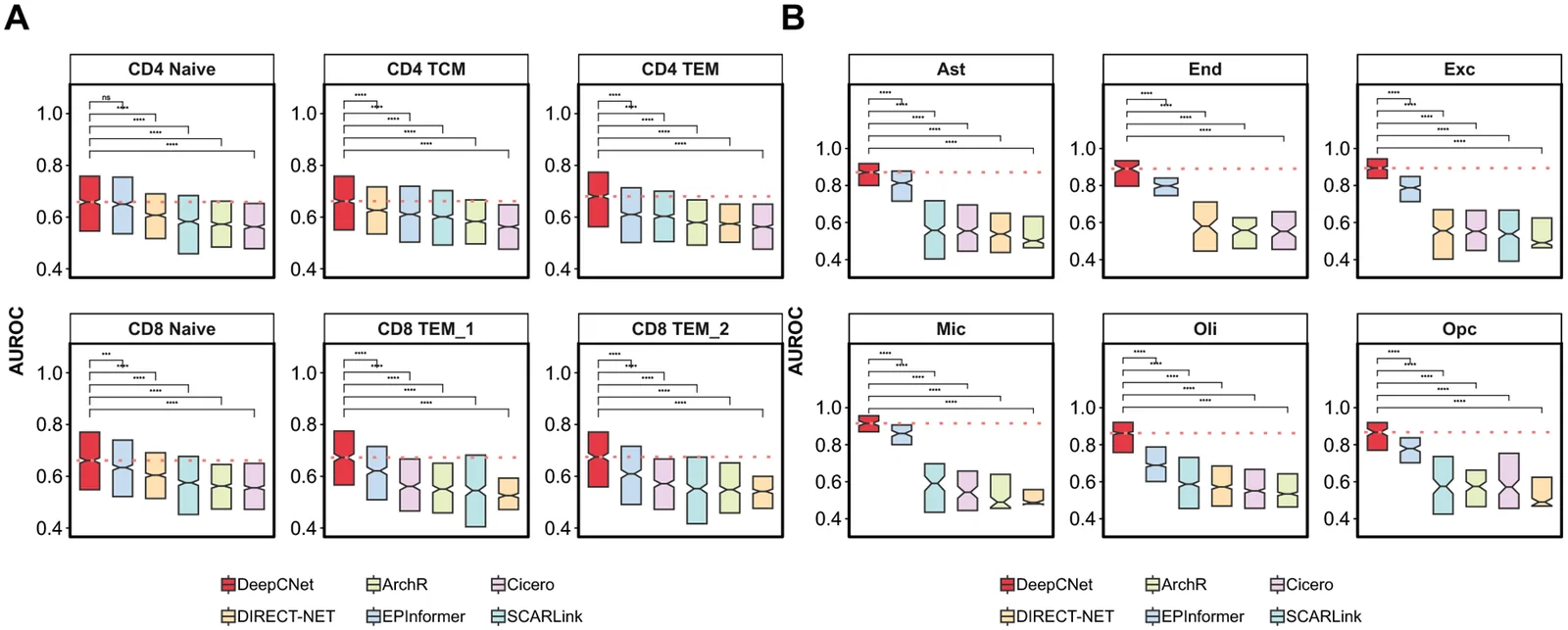
Benchmarking DeepCNet for predicting promoter–enhancer interactions by AUROC. Inferred promoter–enhancer interactions overlapping experimentally validated chromatin interactions were treated as positives, while all remaining interactions were considered negatives. Analyses included only genes satisfying predefined inclusion criteria: more than 10 peaks; more than 5 positives and 5 negatives for the PBMC dataset; more than 3 positives and 3 negatives for the MFC dataset. For each gene, AUROC was averaged across cross-chromosome folds in the training set. Cell types are ranked by the median performance metric values. The AUROC/AUPRC across evaluated genes are visualized by interquartile range (IQR). **A.** AUROC evaluation on the PBMC scMultiome dataset using cell type-matched ChIA-PET data. **B.** AUROC evaluation on the MFC scMultiome dataset using cell type-matched sn-m3C-seq data.

**Figure 4.**
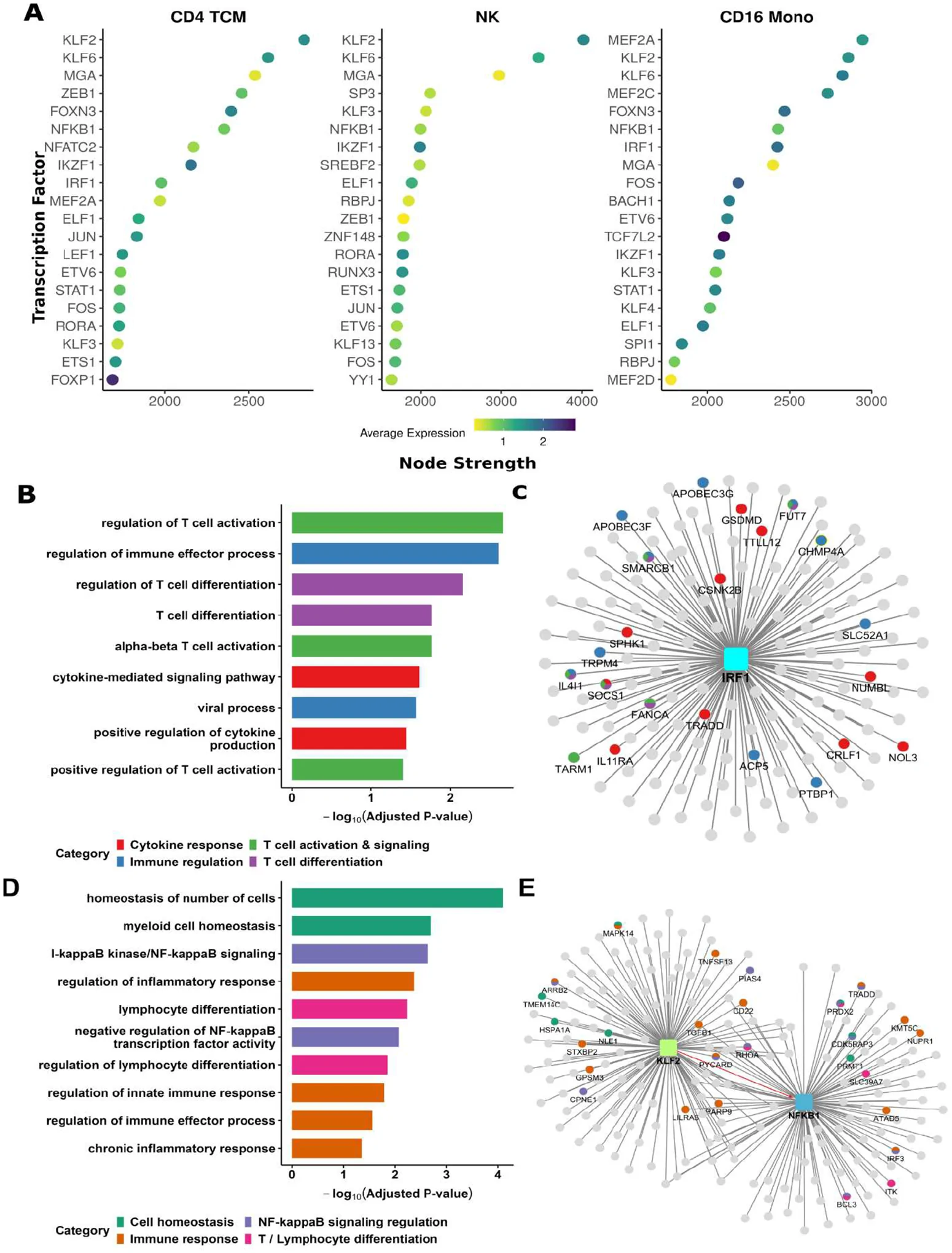
Cell type–specific TF–GRN analysis of PBMC scMultiome dataset. **A.** Top 20 transcription factors (TFs) ranked by node strength in CD4 TCM, NK and CD16 monocytes. **B.** Gene Ontology (GO) enrichment analysis of top 1000 IRF1 target genes in CD4 TCM cells. Bar colors indicate GO term category. **C.** IRF1-centered TF–GRN in CD4 TCM cells, showing top 150 regulatory edges. Node pie charts indicate the GO term categories associated with target genes, with colors corresponding to the GO term categories shown in panel **B. D.** GO enrichment analysis of target genes from top 1,500 KLF2 and NFKB1 regulatory edges. Bar colors denote the GO term categories. **E.** Interaction network between KLF2 and NFKB1 in CD4 TCM cells, showing top 150 regulatory edges for each TF. Node pie charts represent GO term categories of target genes, with colors consistent with the GO term categories shown in panel **D.** Red arrow indicates the interaction from KLF2 to NFKB1.

**Figure 5.**
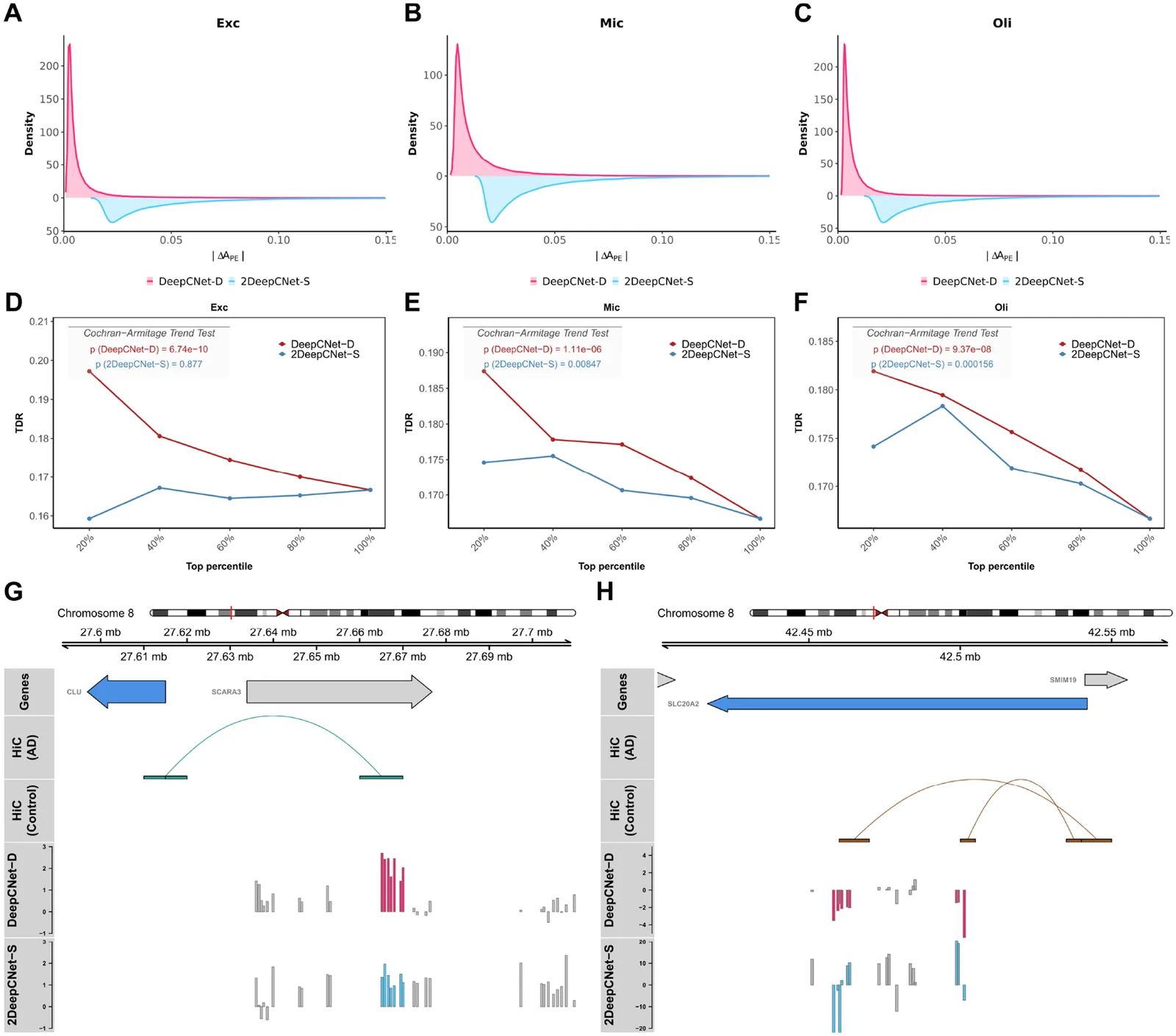
Evaluation of DeepCNet in identifying alteration of promoter-enhancer interactions using DeepCNet. **A-C.** Density plots of the absolute values of DeepCNet-inferred differential PE attention scores ($\Delta {\text{A}}_{\text{PE}}$) between the AD and the healthy control in three cell types: excitatory neurons (Exc), microglia (Mic), and oligodendrocytes (Oli). Top 20% of absolute $\Delta {\text{A}}_{\text{PE}}$ values are shown. Red curves correspond to results obtained from a single joint model (DeepCNet-D), whereas blue curves represent results from modeling the two conditions separately (2DeepCNet-S). **D-F**. Enrichment plots showing the true discovery rate (TDR) of top inferred differential PE interactions validated by two-condition Hi-C loops in the same three cell types (Exc, Mic, and Oli). P-values are calculated using a one-sided Cochran-Armitage trend test across five percentiles (20th, 40th, 60th, 80th, and 100th). **G-H.** Genomic track plots for two genes, CLU and SLC20A2, illustrating condition-specific Hi-C loops and corresponding differential PE attention scores. “DeepCNet-D” and “2DeepCNet-S” track display the differential PE attention scores for each inferred PE interaction (× 10^−3^).

**Figure 6.**
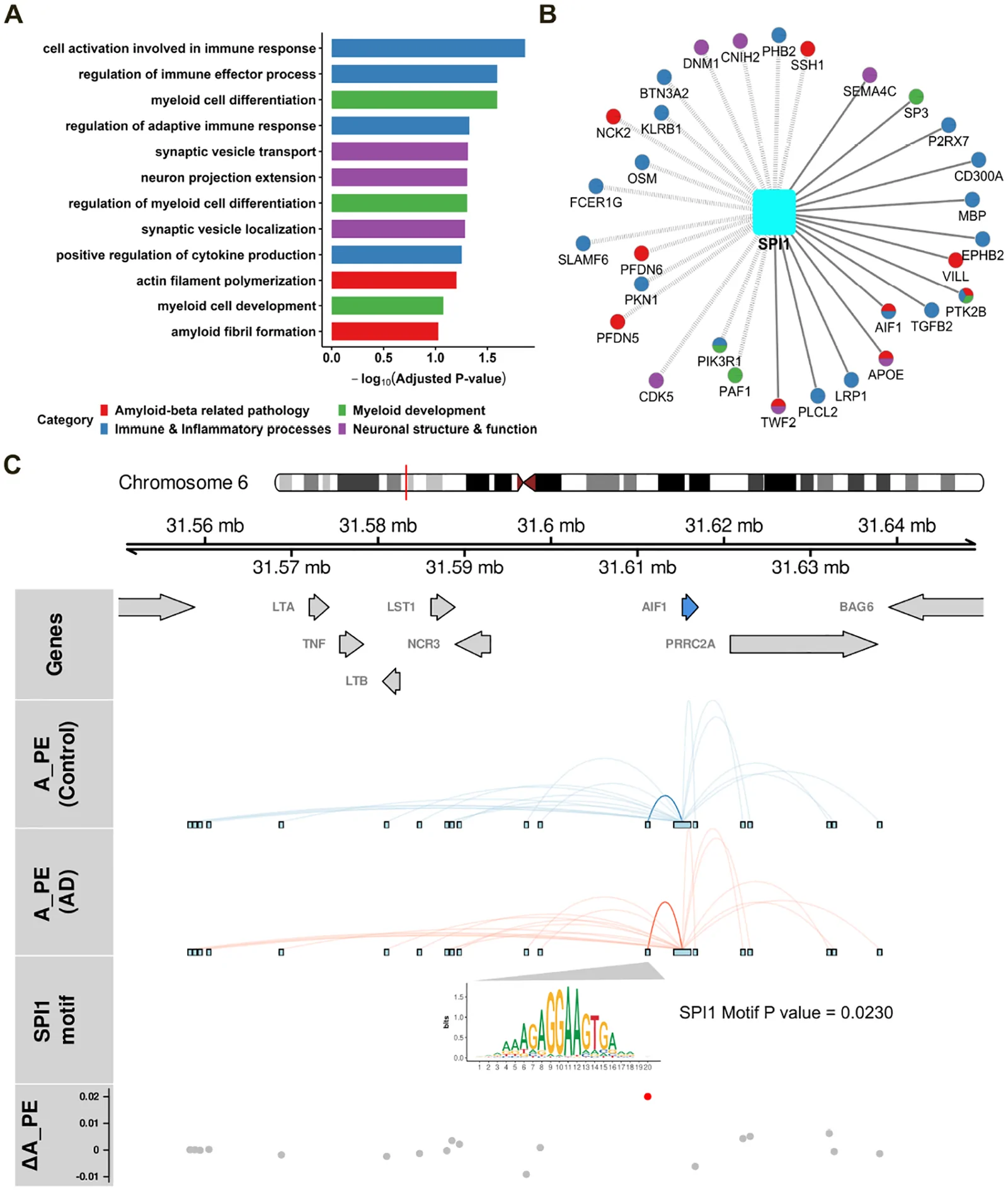
Cell type–specific differential TF-GRN analysis in MFC single-cell multiome data using DeepCNet. **A.** GO enrichment analysis of SPI1 target genes in microglia, using the top 1000 genes ranked by differential TF-gene edge weights. **B.** SPI1-centered differential TF-GRN between the AD and the healthy control in microglia, showing the top 30 genes within GO categories shown in Panel A. Solid edges indicate interactions strengthened in AD, whereas dashed edges indicate interactions weakened in AD relative to the healthy control. **C.** Genomic track plot illustrating condition-specific differences in the SPI1-AIF1 regulatory linkage between AD and healthy control in microglia. “A_PE (AD)” and “A_PE (Control)” tracks show mean PE attention scores across 50 independent runs for interactions between the AIF1 promoter and surrounding ATAC peaks in AD and healthy control, respectively, with loop height representing attention magnitude. “SPI1 motif” track shows the SPI1 motif and its associated motif p-value at the peak of interest. “△A_PE” track shows the mean DeepCNet-inferred differential PE attention scores across 50 independent runs, with the red point highlighting the peak of interest.

## Data Availability

The single cell multiome data for PBMC are available at 10X Genomics website. The human AD single cell multiome data are available at https://personal.broadinstitute.org/bjames/AD_snATAC/. The ChIA-PET chromatin interaction loop files are available from the ENCODE portal. Chro matin contact maps generated by sn-m3C-seq are available at https://salkinstitute.app.box.com/s/fp63a4j36m5k255dhje3zcj5kfuzkyj1. Hi-C data from an AD study profiling from post-mortem p refrontal cortex tissues from both dementia-free elderly individuals and patients with AD are available at http://menglab.pub/hic/. The CRISPR dataset generated in the K562 cell line from ENCO DE is available at https://www.encodeproject.org/files/ENCFF968BZL/. The CRISPRi dataset ge nerated in human primary astrocytes from AstroREG is available at https://github.com/Voineagulab/astrocyte_crispri.
